# Clinicopathological and prognostic significance of SOX9 expression in gastric cancer patients: A meta-analysis

**DOI:** 10.1097/MD.0000000000030533

**Published:** 2022-09-16

**Authors:** Qian Wang, Hao Chen, Congying Yang, Yi Liu, Feng Li, Chunfang Zhang

**Affiliations:** a Department of Pathology, Xuzhou Medical University Affiliated Lianyungang Hospital, Lianyungang City, Jiangsu Province, China; b Department of Pathology and Medical Research Center, Beijing Chaoyang Hospital, Capital Medical University, Beijing, China.

**Keywords:** clinicopathological features, gastric cancer, meta-analysis, prognosis, SOX9

## Abstract

SOX9 is a potential prognostic marker in gastric cancer (GC) patients. This meta-analysis aimed to highlight the clinicopathological and prognostic implications of SOX9 expression in GC patients.

**Methods:**

A systematic literature search was conducted to identify relevant studies by the electronic literature databases (PubMed, Web of Science, EMBASE and Chinese databases). Review Manager version 5.4 was employed to evaluate the pooled odds ratio (OR) or hazard ratio (HR) with 95% confidence intervals (CIs).

**Results:**

Seventeen studies with a total of 2893 GC patients were enrolled in this meta-analysis. The analysis with ten articles clarified that higher expression of SOX9 was observed in GC cancers than that of normal gastric samples (OR = 16.26; 95% CI: 8.16 to 32.42; *P* < .00001). Consequently, the results also showed that SOX9 expression was closely associated with age (OR = 1.34; 95% CI: 1.04–1.72; *P* = .03), tumor size (OR = 0.67; 95% CI: 0.49–0.91; *P* = .01), histological differentiation (OR = 0.62; 95% CI: 0.36–1.06; *P* = .002), tumor stage (OR = 0.48; 95% CI: 0.20–1.12; *P* = .04), lymph node metastasis (OR = 0.36; 95% CI: 0.19–0.67; *P* = .0010) and advanced TNM stage (OR = 0.46; 95% CI: 0.30–0.70; *P* = .0003), but not significantly related to gender, distant metastasis and vascular invasion. Furthermore, high SOX9 expression could significantly indicate poorer overall survival (OS) (HR = 1.40; 95% CI: 1.14–1.72; *P* = .001).

**Conclusion:**

SOX9 overexpression might be related to poor prognosis and could serve as a potential predictive marker of poor clinicopathological prognosis factor in GC patients.

## 1. Introduction

Gastric cancer (GC), with over 1 million new cases and estimated 783,000 deaths worldwide in 2018, ranks the sixth most frequently diagnosed cancer type and the third in the leading cause of cancer death.^[1]^ High incidence and mortality for GC mainly exist in East Asia, Eastern Europe, and South America.^[2]^ The rate of 5-year survival ranges from 5 to 69%, depending on the stage of the disease at diagnosis.^[3]^ Despite the rapid development of the relevant diagnosis and treatment methods in recent years, atypical early symptoms, middle-to-late stage diagnosis, high local recurrence rates after surgery, and distant metastasis remain to be the main reasons of poor prognosis in patients with GC. However, the patients diagnosed at an advanced and/or metastatic stage of GC usually missed the chance of surgery, leading to poor prognosis, causing a major burden on families and society.^[4–6]^ Furthermore, some trials showed that perioperative chemotherapy in patients with GC had a significantly higher overall survival (OS) and progression-free survival (PFS) when compared to patients who only had surgery.^[7,8]^ Gastric cancer may be a molecularly and phenotypically highly heterogeneous disease.^[2]^ Therefore, to improve prognosis, it is necessary to identify novel biomarkers for the early detection of GC, along with its prognosis, and risk of metastatic recurrence, to develop individualized treatment strategies.

SOX9 [sex-determining region Y (SRY)-box 9 protein], a high mobility group box transcription factor, plays a key role in regulating cell fate decisions and stem cell maintenance during embryogenesis and adulthood, including the gastrointestinal epithelium.^[9–11]^ Sox9 is a downstream effector and a regulator of the Wnt pathway, which can exert a significant role in carcinogenesis. In addition, the Wnt/SOX9 signaling pathway affects cell proliferation, differentiation, apoptosis, invasion and migration, such as colorectal cancer and stem cells.^[9,12]^ During the past few years, numerous evidence have revealed that SOX9 have oncogenic properties and upregulated expression of SOX9 was correlated with poor prognosis in patients with malignant tumors, including prostate cancer,^[13,14]^ ovarian cancer,^[15]^ breast carcinoma,^[16,17]^ non-small cell lung cancer (NSCLC),^[18,19]^ esophageal cancer,^[20,21]^ colorectal cancer,^[22]^ osteosarcoma^[23,24]^ and glioma.^[25]^ Growing evidence shows that SOX9 is associated with clinical TNM stage and indicates that SOX9 promotes migration, invasion^[26]^ and the EMT process through the Wnt/β-catenin pathway.^[19]^ In contrast, 2 papers evidenced that SOX9 DNA hypermethylation^[27]^ was present and SOX9 was a potential tumor suppressor in cervical cancer.^[28]^ Therefore, the underlying mechanism of SOX9 functions in GC progression as well as biological function remains unclarified. Some publications have showed that elevated expression of SOX9 is related with poor prognosis in patients with GC.^[29,30]^ However, Sun *et al* reported that SOX9 expression was decreased in GC due to promoter methylation and inversely related to the advanced tumor stage, vessel infiltration, and nodal metastasis, but were not interacted with patient prognosis.^[31]^ Besides, Zhang *et al* and Choi *et al* demonstrated that there were no significant correlations between SOX9 expression and age, gender, tumor size, clinical stage, or lymph node metastasis.^[32,33]^ Therefore, the correlation between SOX9 expression and clinicopathological and prognostic value for GC remains uncertain.

Zu et al^[34]^explained the relationship between SOX9 and the prognosis of gastrointestinal cancer by a meta-analysis, which included eleven studies, found no significant association between SOX9 and clinicopathological characteristics of GC (age, sex, differentiation, lymph node metastasis), the conclusions were weakened. In this study, we performed a meta-analysis to get a more comprehensive and precise understanding of the correlation between SOX9 expression and clinicopathological and prognostic value in patients with GC.

## 2. Materials and methods

### 2.1 Ethics statement

Ethics committee or institutional review board was not necessary for this meta-analysis because our analysis has not affected participants directly, and required data were extracted from previous published studies.

### 2.2 Publication search

We performed a thorough search of the following databases for articles published up to December 2020: PubMed, Web of Science, EMBASE, Wan Fang Data and China National Knowledge Infrastructure (CNKI). The following search terms were used: “SOX9” or “RY-box transcription factor 9” and “gastric cancer” or “gastric carcinoma” or “gastric adenocarcinoma”.

### 2.3 Inclusion and exclusion criteria

The included studies in this analysis should satisfy the following criteria: (1) The patients enrolled were confirmed as GC by pathologists. (2) The expression of SOX9 in GCs was detected by immunohistochemistry. (3) Only studies written in English and Chinese were included in this study. (4) The relationship between SOX9 expression, prognosis and clinicopathological parameters in GC patients was investigated. (5) The study provided enough data to allow the estimation of risk ratios (RRs) or odds ratios (ORs) and their 95% confidence interval (CI). (6) None of patients had received radiation therapy or chemotherapy before surgery.

The exclusion criteria were as follows: (1) experimental studies; (2) reviews, comments, conference abstracts, case reports, or letters; (3) the studies with no clinical data and the relationship between SOX9 expression and prognosis; (4) different articles used of the same patient cohort.

### 2.4 Data extraction and quality assessment

The relevant information of all eligible publications was collected carefully and independently by 3 investigators (QW, HC, and CFZ), including the author, publication year, region, number of patients (cases and controls), research technique, cut-off values, survival data (OS and DFS) and clinicopathological parameters. When the survival data was only presented as Kaplan–Meier curves, we digitally estimated and extracted the data from Engauge Digitizer 4.1 software (from https://sourceforge.net/projects/digitizer/). Any disagreement was solved by discussion between the 3 authors (QW, HC, and CFZ) until a consensus decision was reached. We also selected the Newcastle-Ottawa Quality Assessment Scale (NOS) score to evaluate the quality of the included studies.^[35]^ Briefly, the percentage score (PS) of immunoreactive tumor cells was calculated as follows: 0 (0 %), 1 (1–25 %), 2 (26–50 %), 3 (51–75 %) and 4 (76–100 %). The staining intensity (SI) was visually scored and stratified as follows: 0 (negative), 1 (weak), 2 (moderate) and 3 (strong). The immunoreactivity score (IRS) was obtained in some studies by multiplying the percentage and the intensity score.

### 2.5 Statistical methods

This meta-analysis was performed by using Cochrane Review Manager version 5.4 (Cochrane Library). Pooled ORs and its 95% CI were used to evaluate the association between SOX9 expression and clinicopathological factors of GC patients, including the gender (male vs female), age (≧ 60 years vs <60 years), tumor size (<6 cm vs ≧ 6 cm), histological differentiation (moderate-high vs low), tumor stage (T1 + T2 vs T3 + T4), lymph node metastasis (N0 vs Nx), distant metastasis (M0 vs Mx), vascular invasion (yes vs no), and TNM stage (I-II vs III-IV). Moreover, HR with 95% CI was used to evaluated the relationship between SOX9 expression and the prognostic significance. If the survival data were not directly reported, we also estimated and extracted HR from Kaplan–Meier curves by using the Engauge Digitizer 4.1 software. Subsequently, the I^2^ statistical test were performed to analyze the heterogeneity among studies. If the heterogeneity was obvious (I^2^ value > 50% or *P* < .1), the random effects model was appropriate for the current analysis. Otherwise, a fixed-effects model was performed. Sensitivity analysis was used to assess the influence of individual studies on the estimated summary effect. The 2-sided *P*-value < 0.05 was considered statistically significant.

## 3. Results

### 3.1 Study selection and characteristic

**Figure 1.**
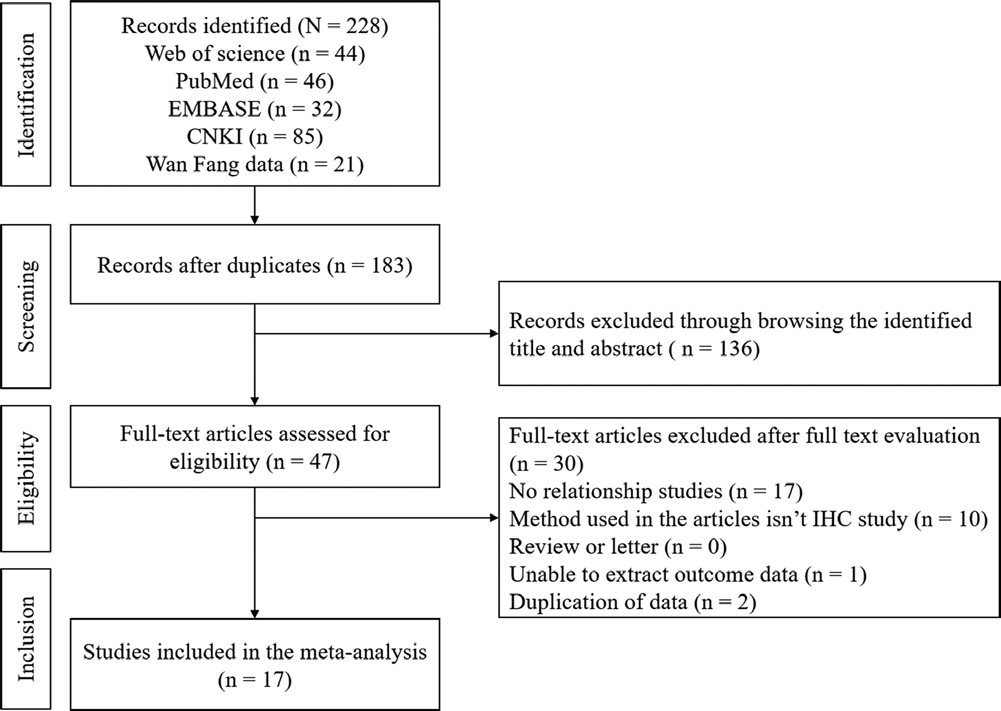
Flow diagram of the procedure for the literature search.

A total of 334 relevant articles were identified on the PubMed, web of science and EMBASE databases, as well as the Chinese databases. After excluding duplication, 75 abstracts were chosen for further evaluation. Subsequently, 18 papers were selected to be read in full. Of these, 1 was excluded for using the same patient cohort. Finally, a total of 17 articles which met the inclusion criteria were considered eligible for the current meta-analysis. The details of selection process were shown in Figure 1.

The main characteristics of the 17 studies were listed in Table 1, including 9 English studies and 8 Chinese studies. All the included studies were published from 2010 to 2020, with all of 3605 sample sizes and 2893 GC patients, and provided the implications of SOX9 expression on the clinicopathological features of GC. Additionally, 9 studies presented survival information (OS and DFS). All of the studies detected SOX9 expression by immunohistochemistry. The characteristics of the included studies are shown in Table 1.

**Table 1:** Characteristics of studies included in this meta-analysis.

Author	Region	Language	Cancer number	Normal cases	Method	Cut-off	Outcomes	NOS score	Ref.
Lei (2020)	China	English	90	90	IHC	IRS > 3	OS	8	^[29]^
Mesquita (2019)	Portugal	English	333	0	IHC	PS > 5%	OS/DFS	8	^[36]^
Li (2018)	China	English	99	0	IHC	SI 2-3	OS	8	^[30]^
Zhang (2018)	China	English	102	40	IHC	IRS > 3	NR	6	^[33]^
Juliana (2016)	Spain	English	76	0	IHC	NR	NR	6	^[37]^
Choi (2013)	Korea	English	185	0	IHC	PS > 30%	OS	8	^[32]^
Sun (2012)	China	English	382	0	IHC	IRS > 5	OS	8	^[31]^
Liu (2012)	China	English	155	18	IHC	PS > 33%	NR	6	^[38]^
Zhou (2011)	China	English	186	0	IHC	PS > 33%	NR	6	^[39]^
Zhang L (2020)	China	Chinese	180	180	IHC	IRS > 6	OS/DFS	8	^[40]^
Zhang X (2020)	China	Chinese	124	40	IHC	IRS > 3	NR	6	^[41]^
Zhu (2020)	China	Chinese	120	120	IHC	IRS > 1	NR	6	^[42]^
Chen (2019)	China	Chinese	70	43	IHC	IRS > 4	OS	8	^[43]^
Liu (2017)	China	Chinese	50	41	IHC	IRS > 3	NR	6	^[44]^
Zhang (2017)	China	Chinese	516	0	IHC	IRS > 4.2	OS	8	^[45]^
Lv (2014)	China	Chinese	113	70	IHC	NR	NR	6	^[46]^
Shao (2012)	China	Chinese	112	70	IHC	IRS > 3	OS	8	^[47]^

IRS = immunoreactive score, IS = staining intensity, NR = not reported, PS = percentage score.

### 3.2 The association between SOX9 levels and the clinicopathological characteristics of GC patients

**Figure 2.**
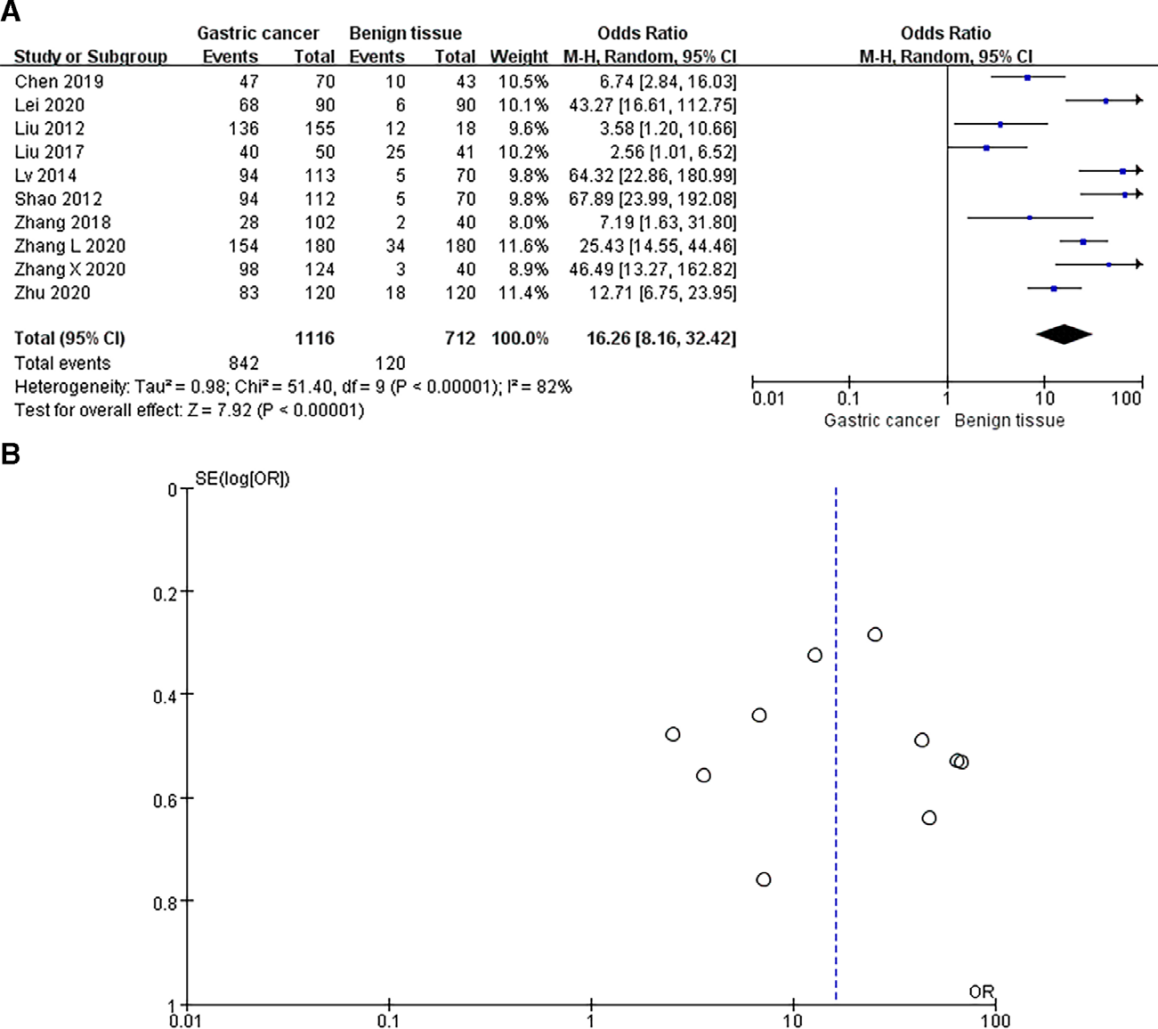
Pooled analysis for the association between SOX9 expression in GC and normal tissue. (A) Forest plots and (B) Funnel plot of publication bias. CI: Confidence interval; OR: Odds ratio.

**Figure 3.**
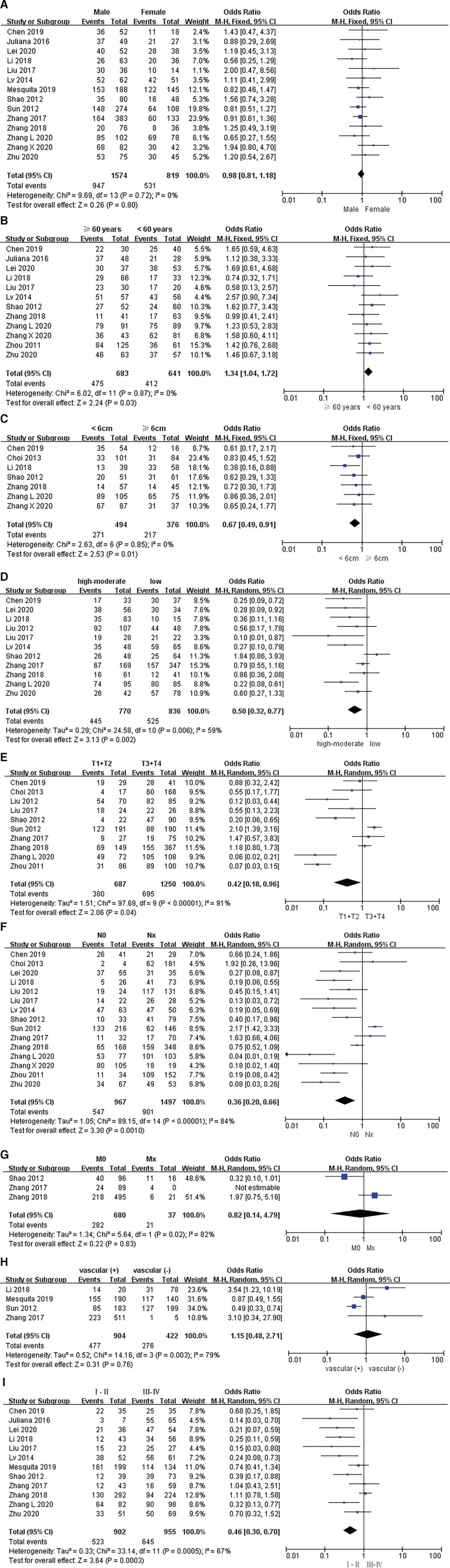
Forest plots for the association between SOX9 expression and clinicopathological features in GC. (A) Gender; (B) Age; (C) Tumor size; (D) Histological differentiation; (E)Tumor stage; (F) Lymph node; (G) Distant metastasis; (H) Vascular invasion; (I) TNM stage.

We explored the correlation between SOX9 expression and clinicopathological features in GC. Ten studies with 1116 GC samples and 712 normal controls demonstrated that SOX9 expression was significantly higher in GC tissues compared with normal gastric tissues (OR = 16.26; 95% CI: 8.16 to 32.42; *P* < .00001; Fig. 2). Seventeen studies with a sample size of 2893 GC patients, summarized the relationship of SOX9 expression and clinicopathological features, and the pooled ORs of SOX9 were listed in Table 2. Twelve studies, including 1324 patients, shown that high SOX9 expression was significantly associated with age (OR = 1.34; 95% CI: 1.04–1.72; *P* = .03; I^2^ = 0%, *P* = .87; Fig. 3B). Moreover, the high SOX9 expression was significantly correlated with the larger tumor size (OR = 0.67; 95% CI: 0.49–0.91; *P* = .01; I^2^ = 0%, *P* = .85; Fig. 3C). Additionally, the high SOX9 expression could significantly predict the poorer histological differentiation in GC patients (OR = 0.62; 95% CI: 0.36–1.06; *P* = .002; Fig. 3D), and the random-effects model was performed due to the significant heterogeneity. Next, our analysis implicated that the overexpression of SOX9 was obviously correlated with tumor stage (OR = 0.48; 95% CI: 0.20–1.12; *P* = .04; Fig. 3E) and lymph node metastasis (OR = 0.36; 95% CI: 0.19–0.67; *P* = .0010; Fig. 3F). More importantly, 12 studies that enrolled 1857 patients demonstrated that high SOX9 expression was significantly associated with more advanced TNM stage (OR = 0.46; 95% CI: 0.30–0.70; *P* = .0003; Fig. 3I). However, significant heterogeneity was observed among those studies, including tumor stage (I^2^ = 91%; *P* < .0001), lymph node metastasis (I^2^ = 84%; *P* < .0001) and TNM stage (I^2^ = 67%; *P* = .0005). However, there was no significant relationship between SOX9 expression and gender (OR = 0.98; 95% CI: 0.81–1.18; *P* = .80; Fig. 3A), distant metastasis (OR = 0.84; 95% CI: 0.28–2.47; *P* = .75; Fig. 3G) and vascular invasion (OR = 1.15; 95% CI: 0.48–2.71; *P* = .76; Fig. 3H).

**Table 2:** Meta-analysis of SOX9 expression and clinicopathological features in gastric cancer.

Clinicopathological features	Study (n)	Cases	Analytical model	Pooled OR (95% CI)	*P* value	Heterogeneity	
						I^2^ (%)	*P* value
Gender (male vs female)	14	2393	Fixed	0.98	0.80	0	0.72
Age (≥60 vs <60)	12	1324	Fixed	1.34	0.03	0	0.87
Tumor sizes (<6 vs ≥6 cm)	7	870	Fixed	0.67	0.01	0	0.85
Grade of differentiation (moderate-high vs low)	11	1606	Random	0.50	0.002	59	0.006
Tumor stage (T1 + T2 vs T3 + T4)	10	1937	Random	0.48	0.09	91	<0.00001
Lymph nodes (N0 vs Nx)	15	2464	Random	0.36	0.001	85	<0.00001
Distal metastasis (M0 vs Mx)	3	730	Random	0.84	0.75	65	0.06
Vascular invasion (- vs +)	4	1326	Random	1.15	0.76	79	0.003
TNM stage (Stage I–II vs III–IV)	12	1857	Random	0.46	0.0003	67	0.0005

CI = confidence interval, Fixed = fixed-effects model, OR = odds ratio, Random = random-effects model.

### 3.3 The prognostic value of SOX9 expression for GC patients

**Figure 4.**
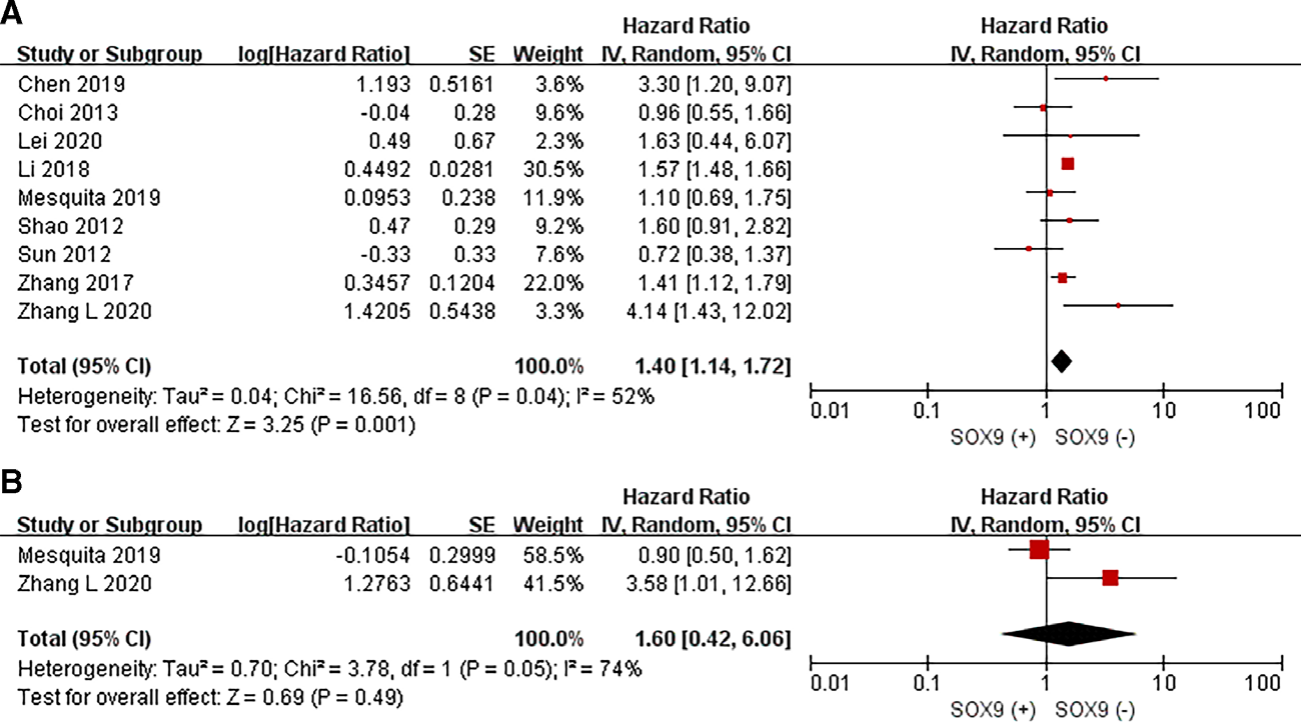
Pooled analysis for the association between SOX9 expression and the survival in GC. (A) Overall survival (OS); (B)Disease-free survival (DFS).

Nine studies with a total of 1911 GC patients were analyzed for prognostic value of the SOX9 expression (Fig. 4). A significant positive correlation between overexpressed SOX9 and poorer overall survival (OS) was observed in the GC patients (HR = 1.40, 95% CI: 1.14–1.72; *P* = .001) in the random effects model with a significant heterogeneity (I^2^ = 52%, *P* = .04). Among the 9 studies on OS, only 4 studies directly provided the multivariable HR, while we evaluated the results from the KM curves in the remaining 5 studies. The results are presented in Table 3. Subsequently, 2 studies evaluated the DFS, the pooled HR was 1.60 (95% CI: 0.42–6.06, *P* = .49; I2 = 74%, *P* = .05) in patients with GC for DFS.

**Table 3:** The prognostic value of SOX9 expression for overall survival in gastric cancer.

Author	HR	Lower limit	Upper limit	Method	Survival	Conclusion
Lei(2020)	1.63	0.44	6.07	Survival curve	OS	Poor
Mesquita (2019)	1.10	0.69	1.75	Survival curve	OS	Unfavorable
Li (2018)	1.57	1.48	1.66	Multivariate	OS	Poor
Choi (2013)	0.96	0.55	1.66	Survival curve	OS	NS
Sun (2012)	0.72	0.38	1.37	Survival curve	OS	NS
Zhang L (2020)	4.14	1.43	12.02	Multivariate	OS	Poor
Chen (2019)	3.30	1.20	9.07	Multivariate	OS	Poor
Zhang (2017)	1.41	1.12	1.79	Multivariate	OS	Poor
Shao (2012)	1.60	0.91	2.82	Survival curve	OS	Poor
Overall	1.40	1.14	1.72	Random		Poor

HR = hazard ratio, NS = not significant, OS = overall survival, Random = random-effects model.

### 3.4 Sensitivity analysis

The sensitivity analysis was performed to test for bias introduced by the low number of available eligible publications in the OS analysis. We excluded the article one by one for sensitivity analysis. The results indicated that the corresponding pooled HRs were not essentially altered by the subtraction of any study (Table 4), revealing that our results were statistically robust.

**Table 4:** Sensitivity analysis for overall survival.

Study omitted(year)	OS HR (95% CI)	I^2^%	Statistical method	*P* value
Lei 2020	1.40 (1.12–1.75)	56	Random	0.003
Mesquita 2019	1.55 (1.47–1.64)	47	Fixed	<0.00001
Li 2018	1.35 (1.13–1.60)	49	Fixed	0.0009
Choi 2013	1.55 (1.47–1.64)	48	Fixed	<0.00001
Sun 2012	1.55 (1.47–1.64)	38	Fixed	<0.0001
Zhang L 2020	1.54 (1.46–1.63)	47	Fixed	<0.0001
Chen 2019	1.37 (1.11–1.68)	50	Random	0.004
Zhang 2017	1.42 (1.05–1.91)	55	Random	0.02
Shao 2012	1.39 (1.10–1.76)	56	Random	0.007

Fixed = fixed-effects model, HR = hazard ratio, OS = overall survival, Random = random-effects model.

### 3.5 Publication bias

**Figure 5.**
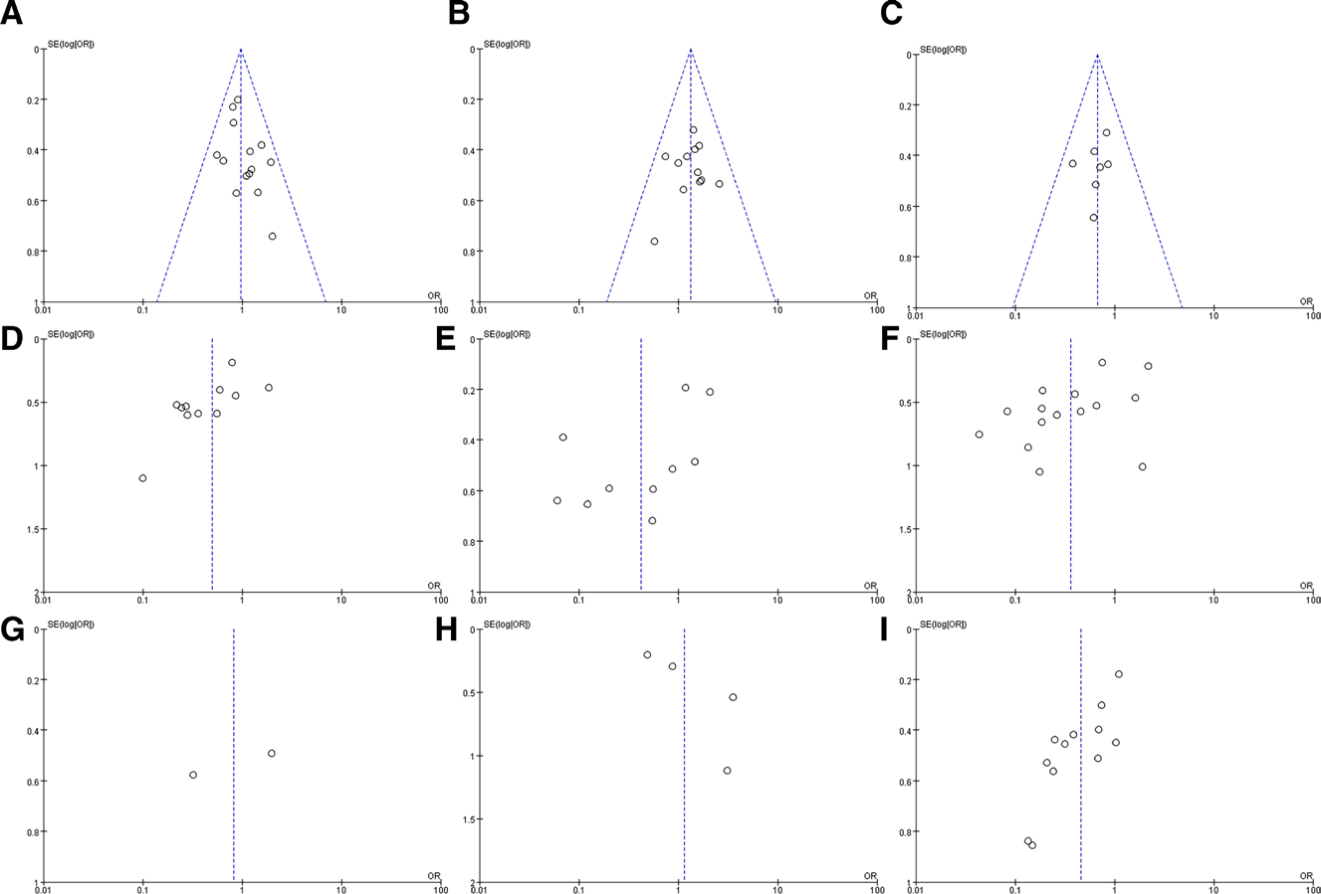
Funnel plots of publication bias for SOX9 expression and clinicopathological parameters in GC patients. (A) Gender; (B) Age; (C) Tumor size; (D) Histological differentiation; (E)Tumor stage; (F) Lymph node; (G) Distant metastasis; (H) Vascular invasion; (I) TNM stage.

**Figure 6.**
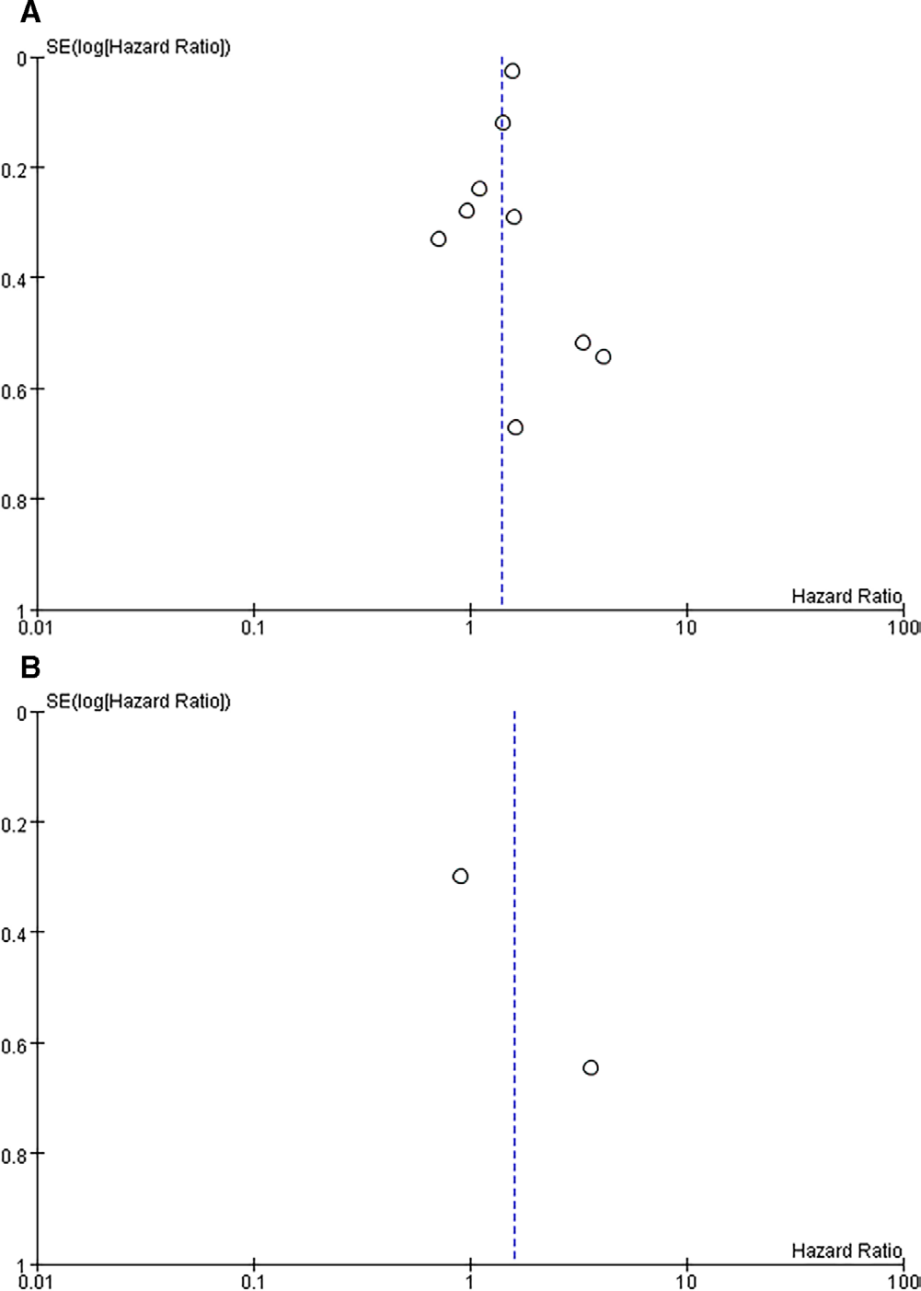
Funnel plots of the publication bias for survival analysis. (A) OS; (B) DFS.

Funnel plot analysis were performed to evaluate the publication bias. As a result, the shape of the funnel plots for the clinicopathological features, OS and DFS revealed no obvious asymmetry. Therefore, there was no obvious publication bias in our meta-analysis (Figs. 5 and 6).

## 4. Discussion

In this study, we performed a meta-analysis to evaluate the clinicopathologic and prognostic significance of SOX9 expression in GC patients. A total of 17 relevant studies comprised of 2756 cases were included to the final analysis. Our results concluded that GC patients with high SOX9 levels had a poor OS compared those with low SOX9 levels, meanwhile, positive SOX9 expression was significantly linked with age, tumor size, histological differentiation, tumor stage, lymph node metastasis and TNM stage.

SOX9, a transcription factor, involved in sex determination, stemness, differentiation, and progenitor development. Previous studies have demonstrated that the SOX9 protein directs pathways involved in tumor initiation, proliferation, migration, metastasis and stem cell maintenance, thereby regulating tumorigenesis as an oncogene. SOX9 elevation could act with WNT signaling to drive cancer progression. And 1 study also shown that SOX9 mediates Notch1-induced mesenchymal features in lung adenocarcinoma.^[15]^ In accordance with its function, large amounts of studies have explored the function of SOX9 expression in hepatocellular carcinoma, breast cancer, prostate cancer, lung cancer, esophageal cancer and colorectal cancer.^[22,48–54]^ Moreover, a previous study found that H. pylori induces SOX9 expression in pretumorigenic gastric mouse cells.^[11]^ Most recently, SOX9 expression also have received widespread attention in GC. The prognostic value of SOX9 expression in GC have been investigated in studies; however, the results are still not consensual. Tingting L *et al* showed that SOX9, a transcription factor, could bind to the COL10A1 promoter, and was essential for COL10A1-mediated EMT, and cell migration, invasion and metastasis.^[30]^ However, Sun *et al* showed that SOX9 downregulation by promoter methylation is related to GC progression, advanced tumor stage, vessel infiltration, and nodal metastasis, but not related to prognosis.^[31]^ To our knowledge, this meta-analysis is the first to evaluate the prognostic and clinical value of SOX9 in GC. Seventeen studies with a total of 1432 patients were enrolled in this meta-analysis, demonstrated that SOX9 expression in GC was significantly higher than that in normal gastric tissues. Then we performed the overall pooled analysis which indicated that positive SOX9 expression was significantly associated with poor OS in GC (HR = 1.4, 95 % CI: 1.14–1.72). Lei and colleagues pointed out that high SOX9 expression have important effects on angiogenesis and are closely related to the poor prognosis of patients with GC.^[29]^ De Lin *et al* reported that SOX9 expression correlates with microvascular density, progress and prognosis in GC patients.^[55]^ Ren *et al*^[56]^ once shown that suppression of Wnt signaling pathway by PPARγ could inhibit its target SOX9 expression in GC cells.

Our results also revealed that SOX9 expression was significantly associated with age, tumor size, histological differentiation, tumor stage, lymph node metastasis and TNM stage, which had the same results in other malignant tumors, such as hepatocellular carcinoma, breast cancer, prostate cancer, lung cancer, esophageal cancer and colorectal cancer.^[21,52,57–60]^ Therefore, it was widely known that SOX9 is able to promote tumor cell proliferation, invasion and metastasis. The present results may explain SOX9 overexpression is associated with poor prognosis in patients with GC, and suggest that SOX9 could contribute to tumor progression in GC. Moreover, it highlights the possible clinical application of SOX9 as an effective therapeutic target in patients with GC.

Although this meta-analysis had investigated the correlation between SOX9 expression and the prognostic and clinicopathological features of GC, some limitations existed in our meta-analysis that should be addressed. First, unpublished studies and abstracts were not enrolled for this analysis, which may result in potential publication bias. Second, the number of included correlated studies is small in this analysis, further study with more enrolled trials are required. Third, the sample sizes of the included studies had no an inclusion criterion, ranging from 50 to 516 patients. Fourth, the protocol and evaluation system to detect SOX9 expression by immunohistochemistry in various studies were uniform, such as differences in types of antibodies, antibody dilutions, and the positive cut-off value were different; these differences may lead to the heterogeneity. Fifth, 5 of 9 studies did not provide HRs and 95% CIs, so estimated data extracted from KM curves may be less reliable than a direct analysis of variance. Moreover, the heterogeneity was high in this analysis. And the source of the heterogeneity was unexplained, the random-effects models are performed.

## 5. Conclusion

In a word, our results are still significant. The high expression of SOX9 was associated with tumor progression and linked with overall survival. Besides, our analysis demonstrated that the strong associations of SOX9 with age, tumor size, histological differentiation, tumor stage, lymph node metastasis and TNM stage in GC patients. overexpressed SOX9 might be served as a potential biomarker for prognostic factors in patients with GC, indicating that directly targeting SOX9 could be potential therapeutic approaches for GC.

## Author contributions

Conception and design: Chunfang Zhang, Feng Li, Hao Chen, Congying Yang and Yi Liu.

Data acquisition: Qian Wang, Hao Chen and Chunfang Zhang.

Data analysis and interpretation: Qian Wang and Chunfang Zhang.

Manuscript drafting: Qian Wang.

Critical revision of the manuscript for scientific and factual content: Chunfang Zhang and Feng Li.

Statistical analysis: Qian Wang and Hao Chen.

Supervision: Chunfang Zhang, Hao Chen, Congying Yang and Yi Liu.

## Acknowledgments

We thank all the participants in this study. This paper is dedicated to all cancer patients.

This work was supported by the Grants from the National Natural Science Foundation of China (nos. 81560399).
